# *SSBP1*-Disease Update: Expanding the Genetic and Clinical Spectrum, Reporting Variable Penetrance and Confirming Recessive Inheritance

**DOI:** 10.1167/iovs.62.15.12

**Published:** 2021-12-14

**Authors:** Neringa Jurkute, Fabiana D'Esposito, Anthony G. Robson, Robert D. S. Pitceathly, Francesca Cordeiro, F. Lucy Raymond, Anthony T. Moore, Michel Michaelides, Patrick Yu-Wai-Man, Andrew R. Webster, Gavin Arno, for the Genomics England Research Consortium

**Affiliations:** 1 Moorfields Eye Hospital NHS Foundation Trust, London, United Kingdom; 2 Institute of Ophthalmology, University College London, London, United Kingdom; 3 Imperial College Ophthalmic Research Unit, Western Eye Hospital, Imperial College Healthcare NHS Trust, London, United Kingdom; 4 Eye Clinic, Department of Neurosciences, Reproductive Sciences and Dentistry, Federico II University, Naples, Italy; 5 Department of Neuromuscular Diseases, UCL Queen Square Institute of Neurology and The National Hospital for Neurology and Neurosurgery, London, United Kingdom; 6 NIHR BioResource - Rare Diseases, Cambridge University Hospitals NHS Foundation Trust, Cambridge Biomedical Campus, Cambridge, United Kingdom; 7 Department of Medical Genetics, Cambridge Institute for Medical Research, University of Cambridge, Cambridge, United Kingdom; 8 Department of Ophthalmology, University of California, San Francisco, San Francisco, California, United States; 9 Cambridge Eye Unit, Addenbrooke's Hospital, Cambridge University Hospitals, Cambridge, United Kingdom; 10 John van Geest Centre for Brain Repair and MRC Mitochondrial Biology Unit, Department of Clinical Neurosciences, University of Cambridge, Cambridge, United Kingdom; 11 North Thames Genomic Laboratory Hub, Great Ormond Street Hospital for Children NHS Foundation Trust, London, United Kingdom

**Keywords:** *SSBP1*, mtSSB, mtDNA replication, inherited optic neuropathy, retinal dystrophy

## Abstract

**Purpose:**

To report novel genotypes and expand the phenotype spectrum of *SSBP1*-disease and explore potential disease mechanism.

**Methods:**

Five families with previously unsolved optic atrophy and retinal dystrophy underwent whole genome sequencing as part of the National Institute for Health Research BioResource Rare-Diseases and the UK's 100,000 Genomes Project. In silico analysis and protein modelling was performed on the identified variants. Deep phenotyping including retinal imaging and International Society for Clinical Electrophysiology of Vision standard visual electrophysiology was performed.

**Results:**

Seven individuals from five unrelated families with bilateral optic atrophy and/or retinal dystrophy with extraocular signs and symptoms in some are described. In total, 6 *SSBP1* variants were identified including the previously unreported variants: c.151A>G, p.(Lys51Glu), c.335G>A p.(Gly112Glu), and c.380G>A, p.(Arg127Gln). One individual was found to carry biallelic variants (c.380G>A p.(Arg127Gln); c.394A>G p.(Ile132Val)) associated with likely autosomal recessive *SSBP1*-disease. In silico analysis predicted all variants to be pathogenic and Three-dimensional protein modelling suggested possible disease mechanisms via decreased single-stranded DNA binding affinity or impaired higher structure formation.

**Conclusions:**

SSBP1 is essential for mitochondrial DNA replication and maintenance, with defects leading to a spectrum of disease that includes optic atrophy and/or retinal dystrophy, occurring with or without extraocular features. This study provides evidence of intrafamilial variability and confirms the existence of an autosomal recessive inheritance in *SSBP1*-disease consequent upon a previously unreported genotype.

Mitochondrial single-stranded DNA-binding protein (mtSSB) is a key element of the mitochondrial DNA (mtDNA) replication machinery, playing an essential role in maintaining mitochondrial function. During replication, the mtDNA unravels and mtSSB, which assembles as a tetramer, selectively binds the exposed heavy strand with high affinity in a noncooperative fashion to prevent nucleolytic attacks, re-annealing and secondary structure formation.^1^^–^^3^ Ineffective mtDNA replication and defective maintenance may lead to point mutations, deletions, and mtDNA depletion causing mitochondrial disease with a range of phenotypes.^4^^,^^5^

Recently, missense variants in *SSBP1* have been reported to cause autosomal dominant mitochondrial optic atrophy and retinal dystrophy with or without mitochondrial depletion.^1^^,^^6^^–^^9^ To date, 16 families (79 affected individual) are reported in the literature with optic atrophy as an unifying clinical feature observed in all affected individuals. Fifty-five percent of examined affected individuals (33/60) showed a variable degree of retinal dystrophy with the majority (23/33 [69.7%]) having a characteristic loss of the outer retina at the fovea. One report includes a singleton case with apparent recessive inheritance of *SSBP1-*associated disease.^7^

This study reports additional autosomal dominant candidate pathogenic *SSBP1* variants and a previously unreported recessive genotype; it also provides detailed clinical and genetic findings that further expand the phenotypic spectrum of *SSBP1*-disease.

## Methods

### Study Cohort

Patients carrying SSBP1 variants were identified by interrogation of whole genome sequencing data as a part of the National Institute for Health Research BioResource—Rare-Diseases and the UK's 100,000 Genomes Project (100KGP) in previously unsolved cases with optic atrophy and/or retinal dystrophy. Four families (two simplex cases and two apparent dominant kindreds) were recruited into the studies through the inherited eye disease clinics at Moorfields Eye Hospital NHS Foundation Trust (London, UK), with an additional UK family identified via the 100KGP recruited at Imperial College Healthcare NHS Trust (London, UK). This study had relevant local and national research ethics committee approvals and adhered to the tenets of the Declaration of Helsinki. Informed consent was obtained from all the patients included in this study.

### Clinical Phenotyping

Retrospective clinical data from the medical records of affected individuals were reviewed after the identification of *SSBP1* variants. All affected individuals underwent an ophthalmological examination during the initial diagnostic workup or after the molecular diagnosis. Electrophysiological testing incorporated the standards of the International Society for Clinical Electrophysiology of Vision and included pattern and flash visual evoked potentials (VEP), and pattern and full-field electroretinography (PERG; ERG).^10^^–^^12^

### Molecular Genetic Analysis

**Figure 1.**
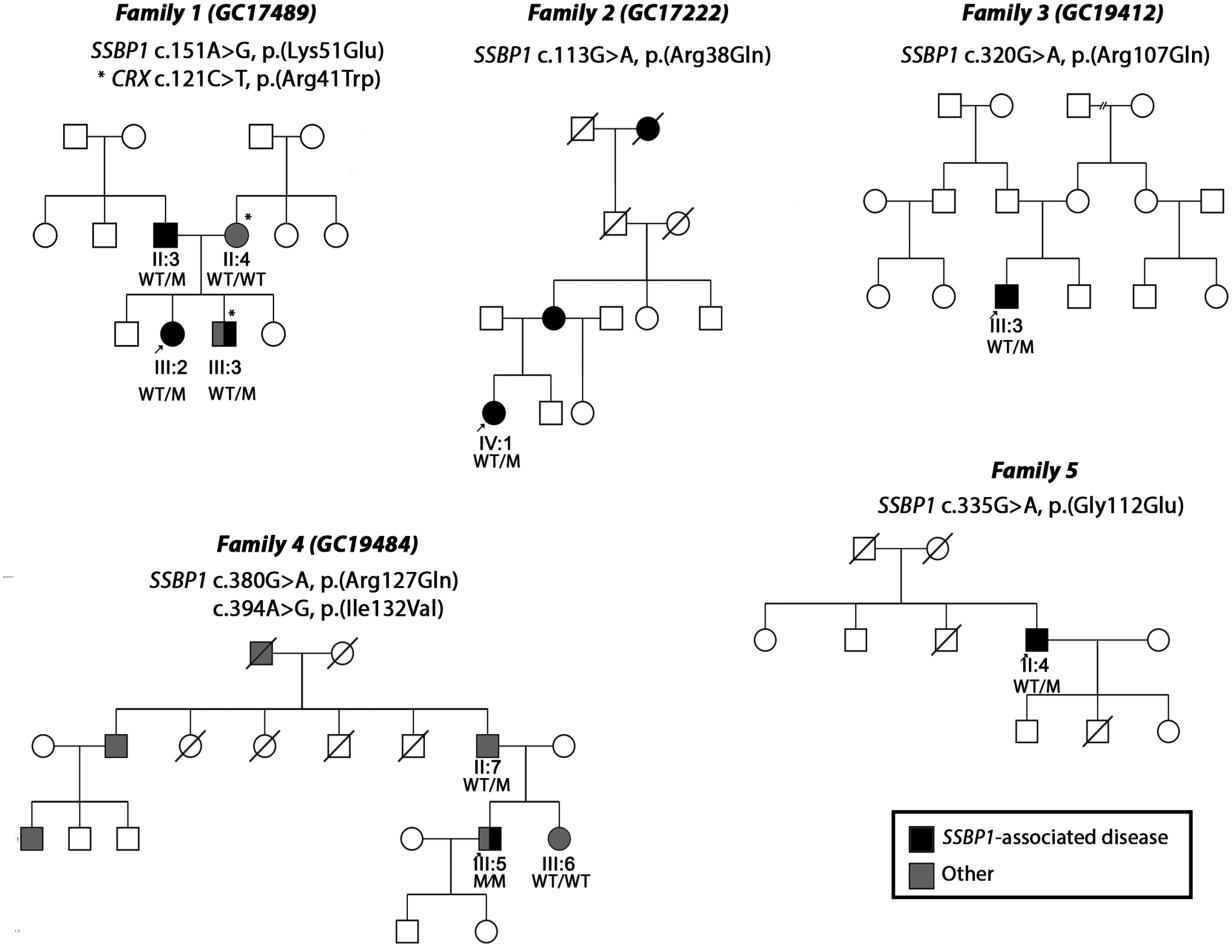
Pedigrees of families 1 through 5 and identified *SSBP1* variants. An arrow indicates proband. Shaded symbols represent affected individuals. Different shading indicates dual pathology: *SSBP1*-disease (black) and other (grey). Asterisk (*) indicates additional pathogenic genotype identified in family 1 (GC17489). M, mutation; WT, wild type.

Whole genome sequencing was performed as part of the National Institute for Health Research BioResource—Rare-Diseases and 100KGP as previously described.^13^^,^^14^ Each affected individual underwent the clinical diagnostic pipeline to identify pathogenic or likely pathogenic variants in a virtual gene panel for posterior segment abnormalities (in-house gene panel or Panelapp Retinal disorders and/or Optic neuropathy, prior to inclusion of *SSBP1* on this panel).^13^^,^^15^ Patients who were negative following this pipeline were selected for further study comprising interrogation for rare (minor allele frequency of ≥0.001) protein-altering *SSBP1* variants in individuals clinically diagnosed with inherited optic neuropathy and/or retinal dystrophy. Variants were confirmed by Sanger sequencing and segregated in family members where available (Fig. 1).

### Bioinformatics

The Genome Aggregation Database (gnomAD, https://gnomad.broadinstitute.org) was used to assess the rarity of variants in the general population. To predict the functional impact of missense variants, in silico prediction tools were applied, including the predictive algorithms of Polymorphism Phenotyping v2 (PolyPhen-2) and Mutation Taster available at http://genetics.bwh.harvard.edu/pph2 and http://www.mutationtaster.org, respectively. Candidate variants were annotated based on American College of Medical Genetics guidelines using the VarSome prediction tool (accessed May 2021).^16^

### Variant Effect on Protein Structure

The evolutionary conservation of the affected amino acid residues across orthologues was assessed using Homologene (https://www.ncbi.nlm.nih.gov/homologene) and sequence alignments were visualized using the Clustal Omega software (https://www.ebi.ac.uk/Tools/msa/clustalo). To assess the predicted effect of the missense variants, the human mitochondrial single stranded binding protein crystal structure (PDB ID: 3ULL and 6RUP) templates from the protein databank (https://www.rcsb.org) were used to model the missense mutant SSBP1 proteins using the Yet Another Scientific Artificial Reality Application (YASARA) software.^17^^–^^19^

## Results

**Figure 2.**
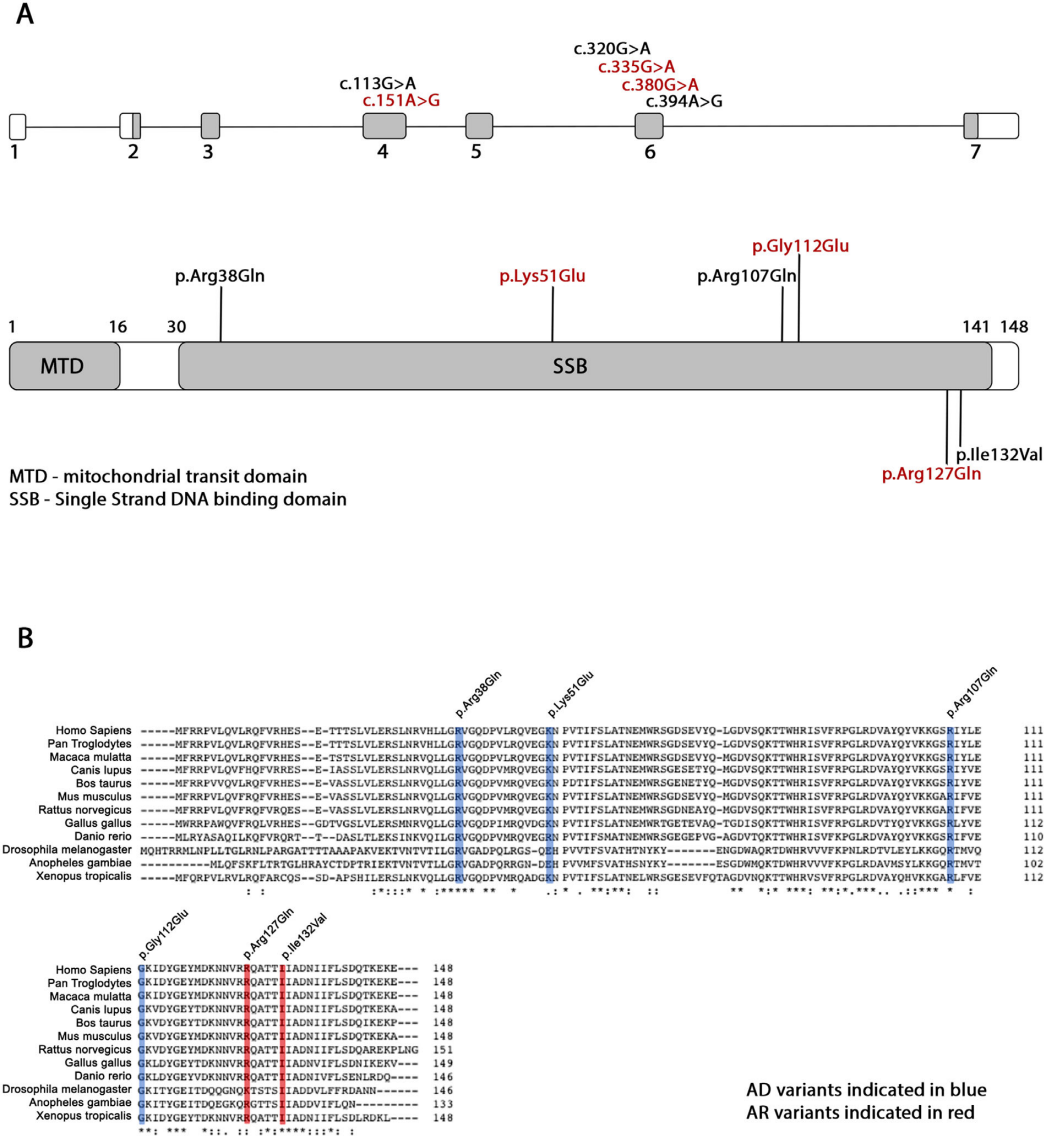
Variant location and conservation. (**A**) Schematic diagram of *SSBP1* gene and SSBP1 protein. The identified variants are indicated on the top at the corresponding exons of the *SSBP1* gene. Autosomal dominant variants are indicated on the top of the bar and autosomal recessive variants are indicated on the bottom of the bar at the corresponding positions of the SSBP1 protein. Novel variants indicated in red. (**B**) Multiple alignment of SSBP1 orthologues. All affected residues are strictly conserved in mammalian orthologues. Autosomal dominant variants are indicated in blue and autosomal recessive variants in red.

We identified seven individuals from five unrelated families (Fig. 1) diagnosed with bilateral optic atrophy and/or retinal dystrophy with extraocular signs and symptoms in some of the affected individuals. Affected individuals from four families were identified to carry heterozygous candidate pathogenic missense variants affecting the SSBP1 protein, with two of them being previously unreported (Fig. 2, Table 1). One individual was found to carry biallelic variants in *SSBP1*, with one of the variants previously reported in association with autosomal recessive *SSBP1*-disease (Table 1).

**Table 1.** *SSBP1* Variants Identified in the Current Study

Family	HGVs	HGVp	Inheritance	gnomAD (MAF)	In Silico	ACMG (accessed May 2021)	Variant Reported	Total Number of Families Carrying Variant (Current Study and Reported)
Family_1	c.151A>G	p.(Lys51Glu)	AD	–	Disease causing	Likely pathogenic	This study	1
GC17489						(PM2, PP1, PP2, PP3)		

Family_2	c.113G>A	p.(Arg38Gln)	AD	–	Disease causing	Pathogenic	Jurkute et al. (2019)	5
GC17222						(PS3, PM1, PM2, PP2, PP3)	Piro-Megy et al. (2020)	

Family_3	c.320G>A	p.(Arg107Gln)	AD	–	Disease causing	Pathogenic	Jurkute et al. (2019)	7
GC19412						(PS3, PM1, PM2, PM6, PP2, PP3)	Del Dotto et al. (2020) Piro-Megy et al. (2020) Lee et al. (2021)	

Family_4 GC19484	c.380G>A	p.(Arg127Gln)	AR	0.00003989 (10 al.)	Disease causing	VUS (PM2, PP2, PP3)	This study	1
	c.394A>G	p.(Ile132Val)		0.00001417 (4 al.)	Disease causing	Likely pathogenic	Del Dotto et al. (2020)	2
						(PS3, PM2, PP2, PP3, PP5)		

Family_5	c.335G>A	p.(Gly112Glu)	AD	–	Disease causing	Likely pathogenic	This study	1
						(PM1, PM2, PP2, PP3)		

Mutation nomenclature was assigned in accordance with GenBank Accession number NM_003143.

ACMG, American College of Medical Genetics; AD, autosomal dominant; al., allele; AR, autosomal recessive; MAF, minor allele frequency; VUS, variant of uncertain significance.

### Clinical Examination of Affected Individuals

**Figure 3.**
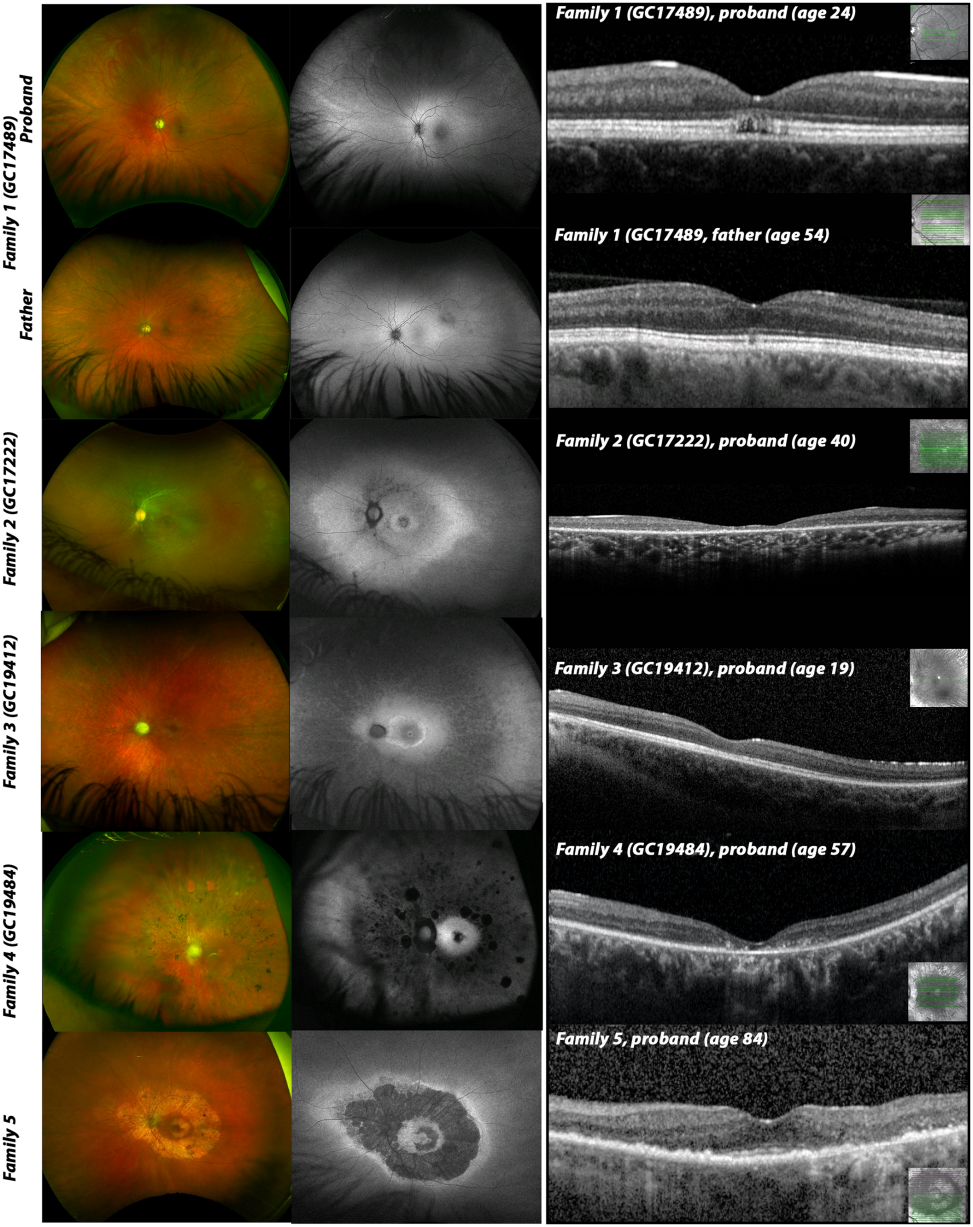
Multimodal imaging composite of affected individuals (left eye only). Optos wide-field fundus imaging (left) shows different degrees of optic atrophy in all affected except II:3 (father, family 1) and proband (family 5). Fundus color photographs demonstrates various degrees of retinal vessels attenuation, pigmentary changes and retinal atrophy in all affected except II:3 (family 1). Optos wide-field fundus autofluorescence images (middle) indicates areas of retinal atrophy with patches of decreased autofluorescence. SD-OCT macula imaging (right) of affected individuals shows various degree loss of inner and outer retina with very mild changes observed in asymptomatic individual (II:3, family 1). The overall retinal thickness is significantly reduced in individuals from family 2, family 3, family 4 and loss of retinal architecture in proband from family 5. Only SD-OCT imaging of proband from family 1 shows signs of the previously described foveopathy, that is, a focal loss of the outer retinal structures.

**Figure 4.**
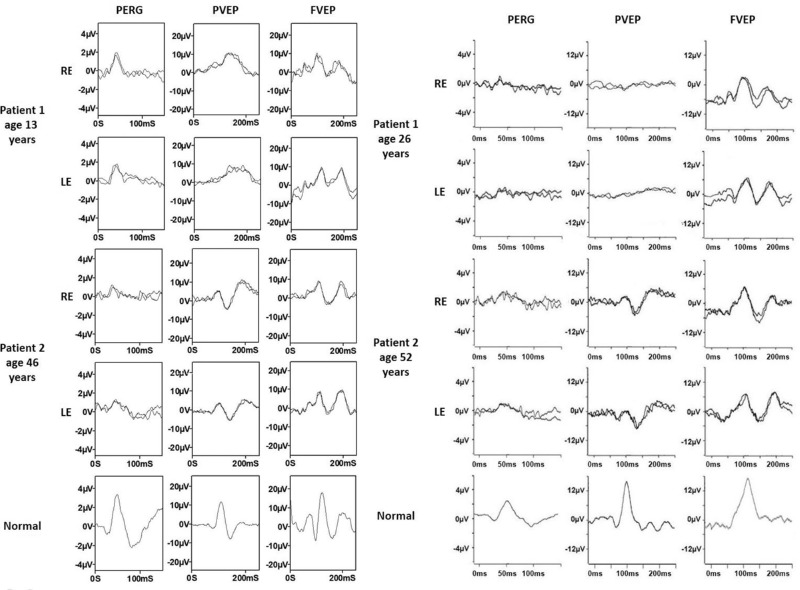
Electrophysiological recordings. International-standard pattern ERG (PERG), pattern and flash VEP (PVEP; FVEP) recordings from family 1 individual III:2 (at ages of 13 and 26 years) and her father II:3 (at ages of 46 and 52 years). Waveforms from right (RE) and left (LE) eyes are superimposed to demonstrate reproducibility. See text for details.

**Figure 5.**
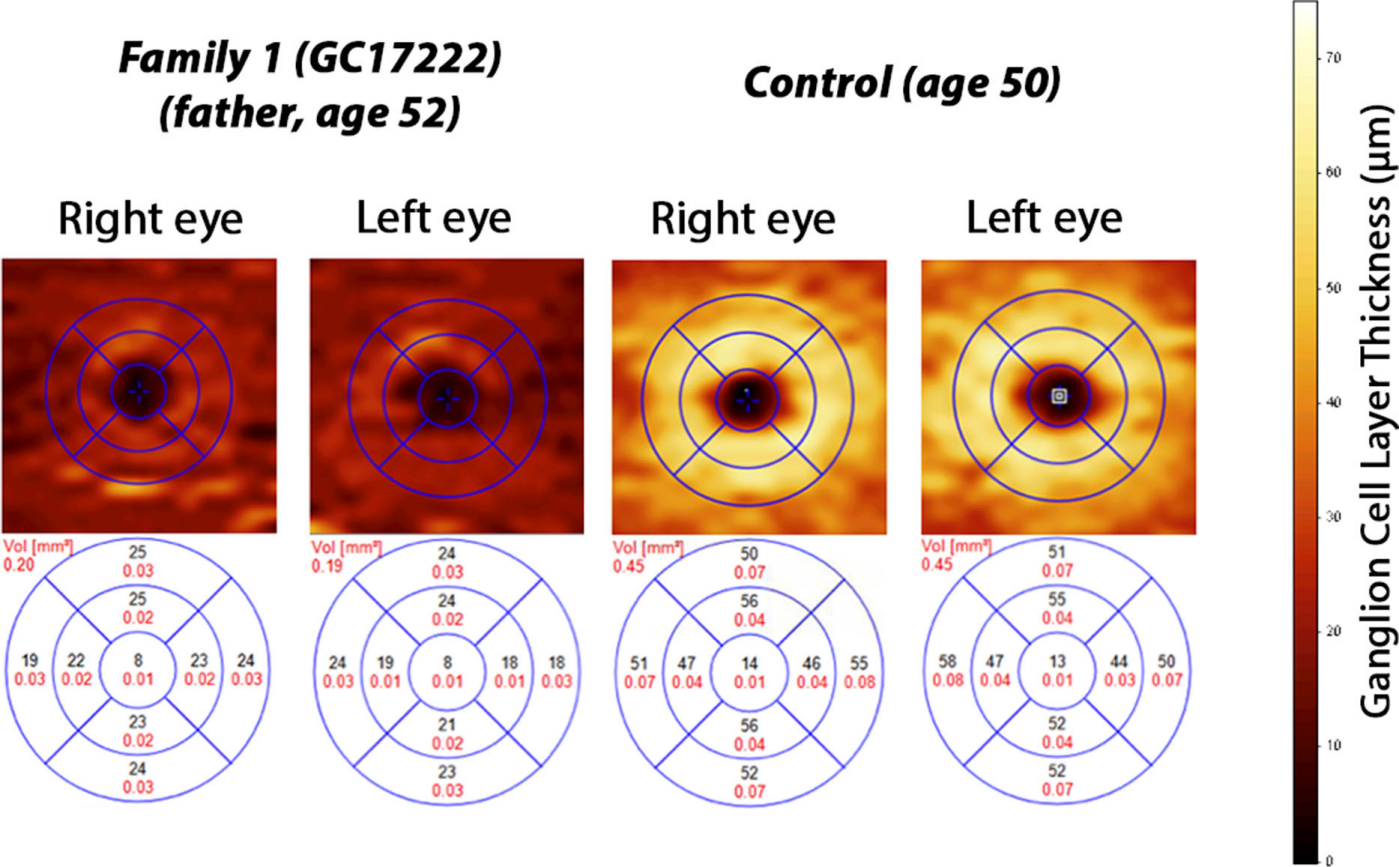
RGC layer thickness maps overlayed with macula grid (1.0, 2.22, and 3.45 mm diameters) on SD-OCT scans in II:3 (family 1) and control. RGC thickness heatmaps indicate RGC layer thinning in II:3 from family 1 (top left panel). For comparison, control RGC thickness heatmaps (top right) illustrates normal RGC layer thickness. Bottom panel show mean volume of RGC layer thicknesses in each of the nine subfields as measured using SD-OCT.

Family 1 (GC17489) was previously investigated and a pathogenic variant c.121C>T, p.Arg41Trp in *CRX* was identified in individual III:3 and his asymptomatic mother (findings reported previously by Hull et al.^20^). Owing to the unusual clinical presentation in III:3 and the nonsegregating optic atrophy and distinct phenotype in III:2, the *CRX* variant was thought to not account for the full phenotype and thus the family was recruited into the 100KGP for further investigation. Individual III:2 was noticed to have vision problems on routine screening at the age of 3 years. At that time, she was diagnosed with bilateral optic atrophy. Her vision significantly deteriorated from the age of 11 years. Later, at the age of 18 to 19 years, she started to experience muscle fatigue, weakness, and intermittent vertigo, which was accompanied by nausea. She also complained of a burning sensation in her hands and feet. At the age of 23 years, she was diagnosed with postural orthostatic tachycardia syndrome. Fundus examination revealed severe bilateral optic atrophy, retinal vessel attenuation, and abnormal foveal reflex. Spectral domain optical coherence tomography (SD-OCT) imaging showed thinning of the retinal nerve fiber layer, retinal ganglion cell (RGC) layer and ellipsoid zone disruption within the fovea region (Fig. 3). Visual electrophysiology at the age of 13 years showed profoundly delayed pattern VEP waveforms that were broadened in shape (peak times of 140 ms on the right and 160 ms on the left; upper limit of reference range, 115 ms). The PERG P50 component was of normal amplitude, excluding a macular cause of pattern VEP abnormality, but P50 was of abnormally short peak time and the N95:P50 ratio subnormal bilaterally, consistent with severe RGC dysfunction bilaterally (Fig. 4). Full-field ERGs were normal, revealing no evidence of generalized rod or cone system dysfunction. Repeat testing at the age of 26 years indicated attenuation of PERG P50, but with a further shortening of peak times bilaterally; pattern VEPs were undetectable, in keeping with progression of optic nerve/RGC dysfunction (Fig. 4). Repeat full-field ERGs were normal. Her father (II:3), 46 years of age at the time of the initial examination, was asymptomatic. His best-corrected visual acuity (BCVA) was 6/12 in the right eye and 6/9 in the left eye. His electrophysiology assessment showed pattern reversal VEPs with a positive peak that was within normal timing limits, but indicated borderline delay on the left compared with the right eye; amplitudes were just within normal limits. The PERG showed abnormalities including a short P50 peak time and subnormal N95:P50 ratio on the right and peak time that was borderline on the left (Fig. 4). There was evidence to suggest additional mild bilateral P50 reduction related to optical factors. Full-field ERGs were normal. At the age of 54 years, his visual function assessment was repeated. His visual acuity remained stable and he was able to read 16/17 and 17/17 Ishihara color tests plates. Visual field testing was within normal limits (data not shown). Fundus examination was within normal limits; however, SD-OCT imaging revealed thinning of the RGC layer (Fig. 5) and disruption of the ellipsoid zone bilaterally (Fig. 3). His repeat visual electrophysiology at the age of 52 years, including ERG, was largely stable, with the exception of a mild shortening of P50 peak time on the left (Fig. 4). These findings suggested bilateral RGC dysfunction, without VEP evidence of optic nerve conduction delay. The index patient's affected brother (III:3) was noticed to have nystagmus and reduced visual acuity at the age of 3 years. At that time, his visual electrophysiology assessment showed delayed flash VEPs and evidence of rod–cone dysfunction on ERG. In addition, he was noticed to have delayed speech and some learning difficulties at secondary school. Over the years, he developed intention tremor and unexplained vertigo, for which he was investigated extensively. He reports that stressful stimuli may lead to fainting, during which he experiences fits. His fundus examination revealed severe bilateral optic atrophy, severe retinal vessel attenuation, mild pigmentary changes, and RPE atrophy. SD-OCT imaging showed significantly reduced retinal thickness. Fundus imaging is available in a previous publication by Hull et al.^20^

The proband from family 2 (GC17222) was born to nonconsanguineous parents from Nigeria. She was 18 years old when deteriorating vision was noticed in both eyes. Her mother and maternal great grandmother had a history of poor night vision and decreased visual acuity from childhood. At the age of 28 years, her visual acuity was 6/36 in the right eye and 6/60 in the left eye. Ophthalmological examination revealed pale optic nerves, mild retinal pigmentary changes in the midperiphery, and retinal vessel attenuation. Fundus autofluorescence imaging showed a hyperautofluorescent ring at the macular and RPE atrophy in the midperiphery and central macular. OCT showed inner and outer retina thinning, with a focal disruption of photoreceptors at the fovea and midperiphery (Fig. 3). The full-field ERGs were in keeping with a rod–cone dystrophy. Pattern ERG P50 components were undetectable, consistent with severe bilateral macular involvement. She was otherwise fit and healthy.

The proband from family 3 (GC19412) experienced a single episode of febrile convulsion at the age of 3 years. At the same age, he was noted to have bilateral nystagmus and reduced visual acuity, which has slowly worsened over the years. He also had developmental delay and attended a special needs school. At the age of 10 years, his ERG assessment showed evidence of a rod–cone dystrophy; pattern VEPs showed marked delays. The same year, he underwent genetic testing using the Asper LCA microarray chip (Apex, Tallinn, Estonia) and direct sequencing of *CRX*, which did not identify any possible cause for the ocular phenotype. His latest BCVA at the age of 16 years was 1/60 in both eyes. Fundus examination revealed pale optic nerves, widespread retinal pigmentary changes, severe retinal vessel attenuation and focal areas of retinal atrophy. OCT imaging indicated both inner and outer retina thinning (Fig. 3).

The proband from family 4 (GC19484) was diagnosed with hearing loss at the age of 2.5 years, which has progressed over time. He was born to nonconsanguineous parents. His father has adolescent-onset hearing impairment of unknown cause and has a family history of hearing impairment consistent with autosomal dominant inheritance. At the age of 15 years, the proband was noticed to have balance problems unrelated to vestibular function, which resolved spontaneously. At that time, he noticed night vision problems, which were followed by peripheral vision loss and at the age of 27 years he was diagnosed with retinitis pigmentosa. At the age of 57 years his BCVA was 6/60 in the right eye and 6/48 in the left eye. Fundus examination showed pale optic nerves with peripapillary atrophy, retinal pigmentary changes in the midperiphery, global retinal atrophy and severe retinal vessel attenuation. OCT imaging indicated both inner and outer retina thinning with loss of the photoreceptor layer (Fig. 3).

The proband from family 5 first complained of nyctalopia at the age of 59 years. His latest BCVA was 6/9.5 in the right eye and 6/7.5 in the left eye. His fundus imaging showed an extensive ring of atrophy at the posterior pole with loss of photoreceptors and RPE. There are signs of preservation of the ellipsoid zone at the fovea (Fig. 3). He was otherwise fit and healthy.

A summary of the ophthalmological and extraocular phenotypic features of the affected individuals has been provided in Table 2, with the retinal findings shown in Figure 3.

**Table 2.** Phenotypic Features Identified in Patients

Family	Individual	Sex	Age of Onset	Latest BCVA	Ocular Phenotype	Visual Electrophysiology (Age at Testing in Years)	Other Signs and Symptoms (Age at Presentation)
Family_1 GC17489	Father (II:3)	M	Adolescence	RE 6/12	Myopia (mild)	RGC/ON dysfunction (46)	–
				LE 6/9		Minimal worsening in LE over 6 years	

	Proband (III:2)	F	Early childhood (3)	RE HM LE HM	Optic atrophy Retinal dystrophy Foveopathy Attenuated retinal vessels	RGC/ON dysfunction (13) Marked worsening over 13 years	Migraine Postural orthostatic tachycardia syndrome (23 years) Fatigue (18–19 years) Anemia Parasthesia in hands and feet Anxiety Vertigo Asthma

	Full-sibling (III:3)	M	Early childhood (3)	RE 3/60 LE 3/60	Optic atrophy Retinal dystrophy^*^ Attenuated retinal vessels Nystagmus	Rod–cone dysfunction	CRX-associated retinal dystrophy Mild learning disability Short-term memory problems Delayed speech Parasthesia in hands and feet Postural tremor Short term memory problem

Family_2 GC17222	Proband	F	Adolescence (18)	RE 3/60 LE 3/60	Optic atrophy Retinal dystrophy Attenuated retinal vessels Myopia	Rod–cone dysfunction Severe macular involvement	–

Family_3 GC19412	Proband	M	Early childhood (3)	RE 1/60 LE 1/60	Optic atrophy Retinal dystrophy Attenuated retinal vessels Myopia (moderate)	ON dysfunction Rod-cone dysfunction	Scoliosis (5 years) Episode of febrile convulsion (3 years) Developmental delay

Family_4 GC19484	Proband	M	Adolescence (15)	RE 6/60 LE 6/48	Optic atrophy Retinal dystrophy Attenuated retinal vessels Early cataracts Myopia (mild)	ND	Hearing loss (2.5 years) Poor coordination, which normalized later (15 years) Coeliac disease (1 year) Speech and language developmental delay

Family_5	Proband	M	Adulthood (59)	RE 6/9.5	Retinal dystrophy	ND	–
				LE 6/7.5			

* Dual retinal dystrophy in affected individual.

HM, hand motions; LE, left eye; ND, no data; ON, optic nerve; RE, right eye.

### Molecular Genetic Analysis

Six *SSBP1* missense variants were identified in seven affected individuals from five families (Table 1). Six individuals harbored heterozygous missense variants likely to be associated with autosomal dominant *SSBP1*-disease. Two of these variants, namely c.113G>A p.(Arg38Gln) and c.320G>A p.(Arg107Gln), have been previously reported in multiple families.^6^^–^^9^

One individual (family 4) had a biallelic genotype with a previously reported variant, c.394 A>G p.(Ile132Val), in *trans* with a rare missense variant, c.380G>A p.(Arg127Gln). For this family, because of the dominant family history of hearing loss, genetic analysis within the 100KGP clinical pipeline was focused initially on candidate autosomal dominant disease variants, which was negative. A review of the clinical history of hearing loss in the family suggested that the proband's hearing loss may have a different etiology to that of his father, and he was the first in the family affected with an ocular phenotype in keeping with *SSBP1*-disease (Fig. 1). Therefore, a reanalysis was performed, additional genes and biallelic variants that may explain the ocular disease and hearing loss were also considered within the optic neuropathy, retinal disorders, and hearing loss gene panels (https://panelapp.genomicsengland.co.uk). We identified the rare *SSBP1* c.380G>A p.(Arg127Gln) variant inherited on the paternal allele which was too common (minor allele frequency 0.00003989, 10 heterozygous alleles) for an autosomal dominant disease variant in *SSBP1*; thus, candidate protein-altering variants on the *trans* allele were investigated. This revealed a single rare variant, c.394A>G p.(Ile132Val), that has been reported previously in *SSBP1*-associated autosomal recessive disease.

All variants were located within the SSB domain (Fig. 2A) and were predicted to be damaging/disease causing by in silico prediction algorithms. All missense variants causing autosomal dominant disease were absent from the gnomAD dataset, whereas variants causing autosomal recessive disease were rare (Table 1). Multiple alignment of SSBP1 orthologues confirmed the strict conservation of the affected amino acid residues across evolution with only Lys51 (differing in 3/12 species) and Arg127 (differing in 1/12 species) exhibiting any divergence (Fig. 2B).

### Prediction of the Effect of SSBP1 Variants on Protein Structure and Interactions

**Figure 6.**
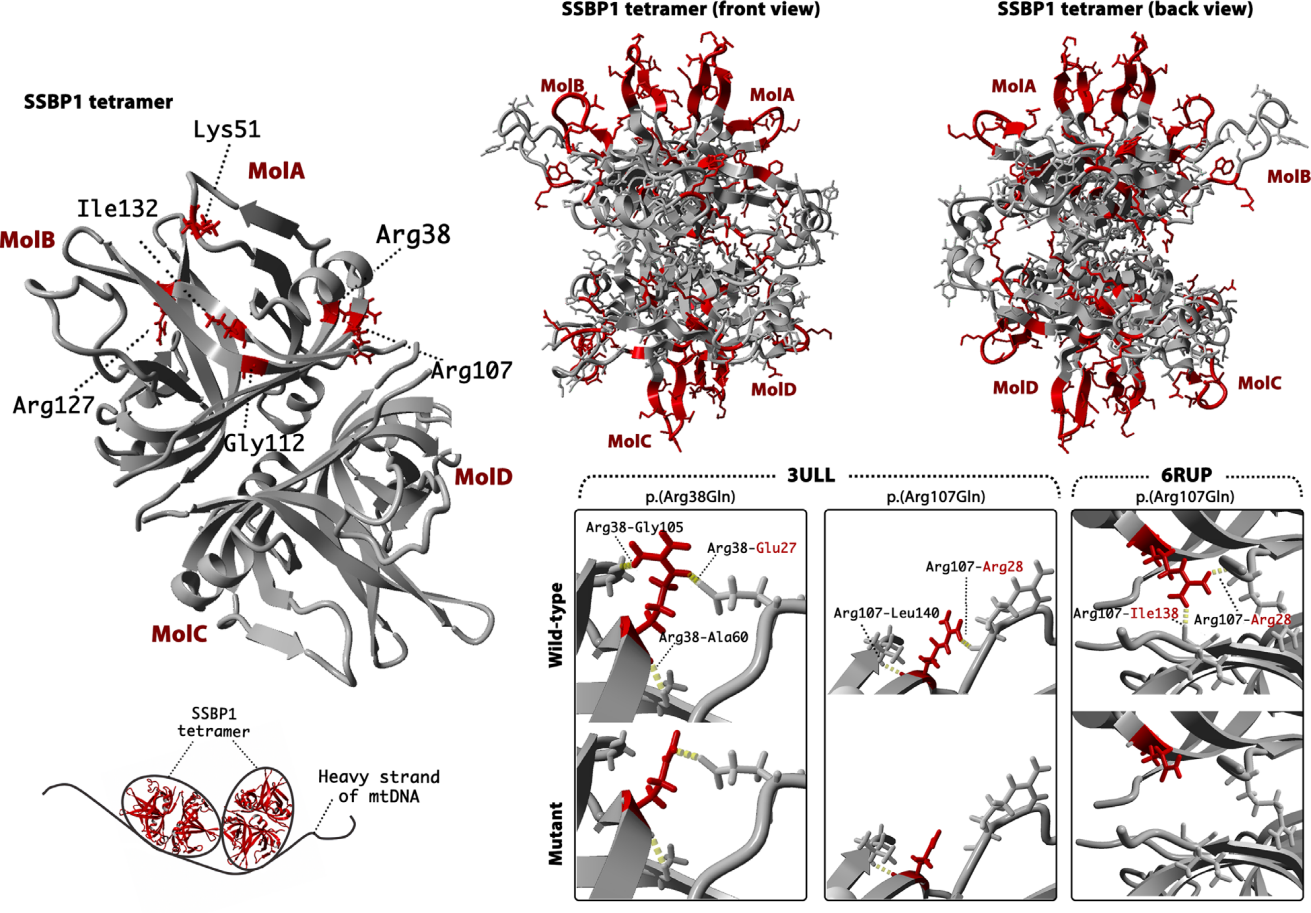
Three-dimensional modelling of SSBP1 using protein databank crystal structures: 3ULL and 6RUP. The tetramer of SSBP1 (top left) was modelled using 3ULL template. Affected residues are indicated in red within molecule A. The bottom left panel is a schematic representation of how the heavy strand of mtDNA wraps around the SSBP1 tetramer based on dual resonance frequency-enhanced electrostatic force microscopy (DREEM) imaging studies by *Kaur et al*.^3^ The top right panel shows residues (red) located within the electropositive patches of SSBP1 as described by Yang et al.^18^ Bottom right panels are zoomed in on the residues that showed an alteration of hydrogen bonds in wildtype (WT) and mutant (M). Mutant modelling was performed using the 6RUP dimer (for Arg38 and Arg107) and 3ULL tetramer (For Arg107). Identified hydrogen bonds (dashed yellow lines) are indicated in both, WT and M molecules, with labelling in WT only. Mutant residues are shown in red. Binding partners are highlighted (black, within MolA; red, MolB or MolD).

**Figure 7.**
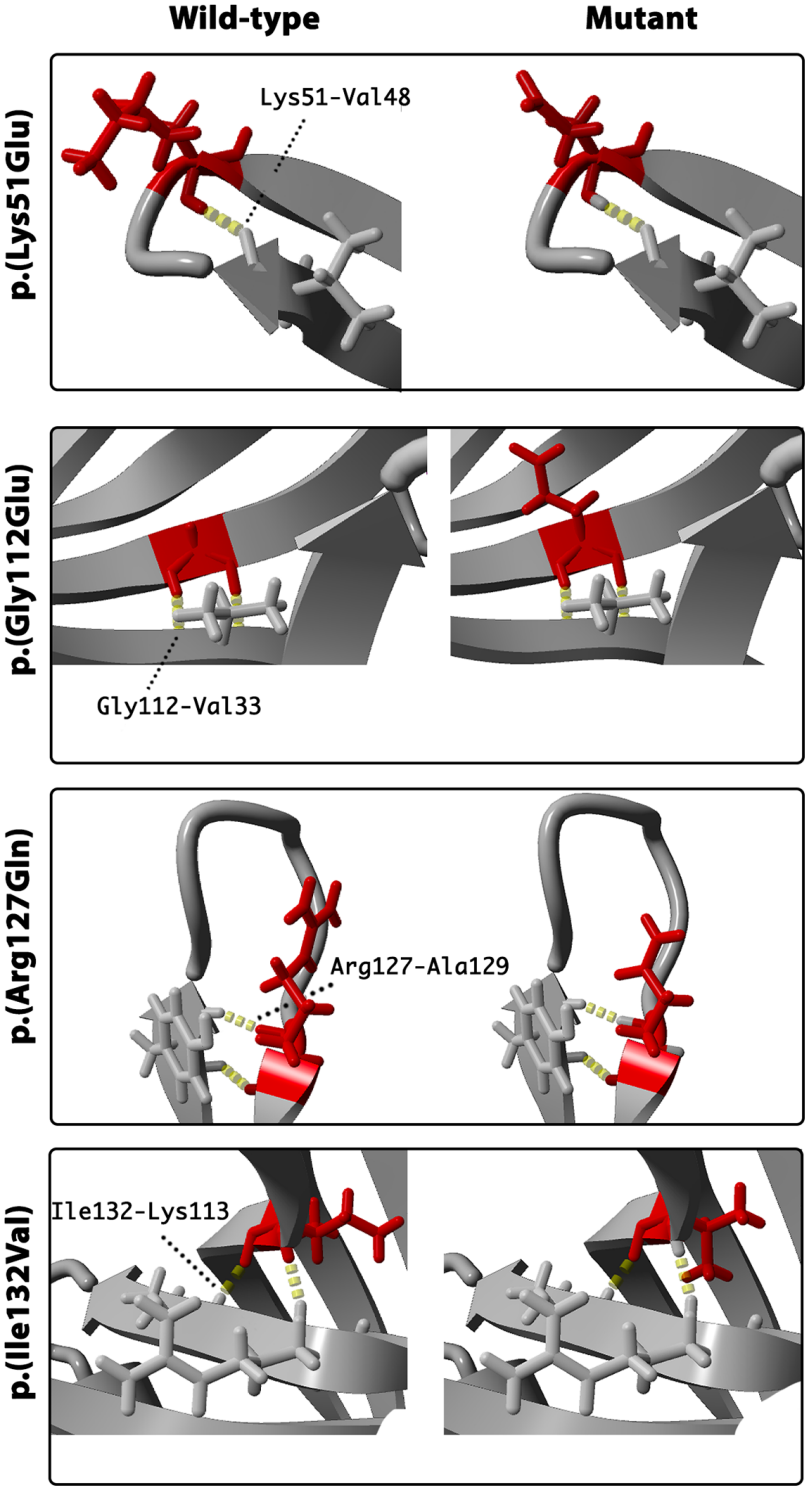
Three-dimensional modelling of SSBP1 using protein databank crystal structure: 6RUP. Each panel demonstrates zoomed residues in wildtype (WT) and mutant (M) for the residues, which did not show altered hydrogen bonds in mutant. Identified hydrogen bonds (dashed yellow lines) are indicated in both, WT and M molecule, with labelling in WT only.

The SSBP1 tetramer is formed by two interacting asymmetric dimers (Fig. 6, left panel). Positions of the identified variants are indicated in red in molecule A. All mutated residues except Lys51 are located within the β-sheet. The latter is found on a flexible loop at the outer surface of the molecule. Hydrogen bonds connect residues Arg38 and Arg107 to binding partners within the dimer and as such possibly help stabilize the dimer structure, with the Arg107 also forming an additional bond (MolA Arg107- MolD Ile138) to form and stabilize the tetramer (Fig. 6, bottom right panel). Hydrogen bonds of residues Lys51, Gly112, Arg127 and Ile132 are located within the monomer. Three-dimensional mutant protein modelling showed loss of hydrogen bonds between different molecules for p.(Arg38Gln) and p.(Arg107Gln) mutants and may, therefore, affect dimer and tetramer assembly. In addition, all residues are located within or close to the protein surface and four in six lead to a loss of positively charged residues in a basic patch (Arg38, Arg107, Lys51, Arg127), which may suggest a possible mechanism of decreased affinity in binding single-stranded DNA (ssDNA). A summary of residues interactions in wild-type, mutated protein, change in charge and a possible effect on the protein in both wild-type and mutant is provided in Table 3, Figure 6, and Figure 7.

**Table 3.** A summary of Residues Hydrogen Bonds in Wild Type and Mutant (Molecule A) and Their Possible Effect on Protein Function and Structure

Disease	Residue	Patch*	MolA	Mutant	Amino Acid Charge Change	Possible Effect on Function and/or Structure of Protein
AD	Arg38	Patch B	MolA Arg38–MolB Glu27 MolA Arg38–MolA Gly105 MolA Arg38–MolA Ala60	HB with Gly105 is lost	From positive to not charged	WT: Direct contact with ssDNA; higher structure formation M: Reduced affinity to bind ssDNA; affected assembly
	Lys51	Patch A	MolA Lys51–MolA Val48	HB remain intact	From positive to negative	WT: Direct contact with ssDNA M: Reduced affinity to bind ssDNA
	Arg107	Patch B	MolA Arg107–MolB Arg28 MolA Arg107–MolD Ile138^†^ MolA Arg107–MolA Leu140	HB with Leu140 and Ile138 is lost	From positive to not charged	WT: Direct contact with ssDNA; higher structure formation M: Reduced affinity to bind ssDNA; affected assembly
	Gly112	Adjacent to patch C	MolA Gly112–MolA Val33	HB remains intact	No change	WT: Unknown M: Unknown

AR	Arg127	Patch A	MolA Arg127–MolA Tyr119	HB remains intact	From positive to not charged	WT: Direct contact with ssDNA; higher structure formation M: Reduced affinity to bind ssDNA; affected assembly
	Ile132		MolA Ile132–MolA Ser88 MolA Ile132–MolA Arg86	HB remain intact	No change	Previous functional assays showed that mutant leads to lower thermostability of the tetramer.

* Electropositive patch as identified by Yang at al.^18^ Patch A consists of Arg46, 46–52, Trp84, Arg86, 119–126 and Arg127 residues; patch B of Arg28, Arg38, Lys104, Arg107 residues; patch C of Trp65, Arg66, Lys81, Phe90, Arg91 and Lys113 residues; and patch D of Lys122 and Arg126 residues.

† HB binding Arg107 with an Ile138 in opposing dimer is observed in tetramer using 3ULL template.

AD, autosomal dominant; AR, autosomal recessive; HB, hydrogen bond; M, mutant; MolA, molecule A; MolB, molecule B; MolC, molecule C; MolD, molecule D; ssDNA, single stranded DNA; WT, wild type.

## Discussion

Here we report the detailed clinical and genetic findings in seven individuals from five unrelated families affected by *SSBP1*-disease. In addition, we report the second case of likely recessive *SSBP1*-disease with the previously unreported variant p.(Arg127Gly) in *trans* with the previously reported recessive allele p.(Ile132Val).

mtDNA maintenance depends on nuclear genes encoding components of the mtDNA replication and its repair machinery. Unsurprisingly, pathogenic variants disrupting these genes result in impaired mtDNA synthesis, which leads to mtDNA maintenance defects and a broad phenotypic spectrum of mitochondrial disease.^5^^,^^21^^,^^22^ MtSSB (SSBP1, encoded by *SSBP1*) is an indispensable component of the mtDNA replication machinery and, as demonstrated previously, defects in SSBP1 may lead to an isolated or syndromic phenotype characterized by optic atrophy and retinal dystrophy.^1^^,^^6^^–^^9^

Mitochondrial diseases are genotypically and phenotypically heterogeneous with ocular (predominantly optic atrophy or progressive external ophthalmoplegia) involvement being a major and frequently debilitating feature. With the latest advance in genomics and access to unbiased genomic sequencing, a growing number of novel gene–disease associations have emerged and retinal degeneration is now increasingly recognized as an important manifestation of the mitochondrial ocular phenotype.^1^^,^^6^^–^^8^^,^^23^^–^^27^ The commonest retinal changes seen in mitochondrial diseases is pigmentary retinopathy with the classical salt and pepper appearance. However, rod–cone dystrophy is an increasingly recognized phenotype and has been additionally reported in patients carrying pathogenic variants in *MT-ATP6* (MIM *516060),^28^ *MT-TS2* (MIM *590085),^29^ and recently in *COQ2* deficiency (MIM *609825),^30^ representing diverse functional pathways within the mitochondrion. The pathogenesis of the retinal degeneration seen with mitochondrial dysfunction could be related to this tissue's high metabolic demand and inadequate energy production or possibly owing to an indirect effect on the photoreceptors.^26^ As previously reported and observed in the current study, optic atrophy and retinal dystrophy are the major clinical features recognized in patients harboring *SSBP1* variants with RGC, photoreceptors and RPE being the site of highest SSBP1 expression.^8^

In our patient cohort, a distinct pattern of photoreceptor and RPE atrophy was observed in the oldest patient (family 5), compared with the retinal dystrophy observed in other affected individuals. In addition, the retinal nerve fiber layer profile in that particular individual remained intact. Taken together, these findings may suggest a different mechanism of disease for this allele or alternatively that the variant is not associated with disease and the causative variant(s) remain to be identified. It is noteworthy that a previously reported *SSBP1* mutation affects the adjacent codon p.(Glu111Gln).^7^

Missense substitution can affect protein function in a number of ways. It is hypothesized that pathogenic *SSBP1* variants lead to altered affinity of the SSBP1 complex to mtDNA or instability of the assembled dimer/tetramer. Interestingly, recent molecular dynamics simulation experiments showed that all previously published variants do not affect SSBP1 tetramer stability in the model system and, as such, this work contradicts the previous tetramer disruption hypothesis.^31^ Yet, residues located on the tetramer surface are involved in ssDNA interaction, and charge alteration at these residues could lead to a decreased affinity to ssDNA. Five distinct binding modes for ssDNA wrapping around the mtSSB have been proposed.^18^ The authors identified electropositive patches A to D (the electrostatic attraction between the negatively charged DNA and positively charged molecules), where an electropositive channel is guided by β-hairpin loops on the surface of the molecule (Fig. 6). Later studies on mtSSB binding kinetics and thermodynamics suggested two binding modes defined by ssDNA binding sites sized 30 and 60 nucleotides, which are modulated by NaCl and Mg^2+^ concentrations.^32^ The authors suggested that ssDNA binding site length determines the ability to bind to one-half or all mtSSB subunits and wrap around the tetramer. A subsequent study used dual resonance frequency-enhanced electrostatic force microscopy imaging to investigate mtSSB–ssDNA complex binding, which revealed a single binding manner in which ssDNA wraps once around mtSSB.^3^

Four variants reported here affect positively charged residues: Arg38, Lys51, Arg107, and Arg127. Both Arg38 and Arg107 are within the electropositive patch B, which is essential in forming the mtSSB–ssDNA complex and a possible mechanism of decreased affinity in binding ssDNA has been suggested previously.^6^^,^^18^ Notably, the residue affected by a novel candidate variant p.(Lys51Glu) is located on the flexible loop within the electropositive patch A.^18^ The alteration from a positively charged lysine to negatively charged glutamate may, therefore, lead to a decreased affinity to bind ssDNA. Strikingly, all variants except p.(Ile132Val) are located within or adjacent to an electropositive patch. Residue Gly112 is adjacent to a lysine at position 113 located within patch C (Table 4). Interestingly, Glu111 and Gly112 are both mutated in *SSBP1*-disease, suggesting this region to be important functionally.^7^ Molecular dynamics simulation studies identified that previous reported pathogenic *SSBP1* variants lead to subtle changes in mtSSB–ssDNA interaction by affecting binding, wrapping, or release, subsequently causing mtDNA maintenance failure.^31^

**Table 4.** Residues Located Within the Electropositive Patches of SSBP1

Patch	Residue Yang et al.	Residue NM_003143.3*	Variant in the Patch/Adjacent to the Patch
	Arg30	Arg46	
	30–37	46–52	
A	Trp68	Trp84	p.(Lys51Glu)
	Arg70	Arg86	p.(Arg127Gln)
	119–126	119–126	
	Arg111	Arg127	

	Arg12	Arg28	
B	Arg22	Arg38	p.(Arg38Gln)
	Lys88	Lys104	p.(Arg107Gln)
	Arg91	Arg107	

	Trp49	Trp65	
	Arg50	Arg66	
C	Lys65 Phe74 Arg75 Lys97	Lys81 Phe90 Arg91 Lys113	p.(Gly112Glu)

D	Lys106	Lys122	p.(Arg127Gln)
	Arg110	Arg126	

* The sequence indicated by Yang et al.^18^ was reviewed and residues were assigned to up to date annotation (NM_003143.3).

Some of the reported variants may be important in both ssDNA binding and dimer/tetramer assembly. Previous studies showed that the inner β-barrel scaffold is preserved and is necessary to ensure the stability at the tetramer interface. The previously reported variants, p.(Arg38Gly) and p.(Arg107Gly), may affect the formation of the higher structure of mtSSB. Both variants affect arginine residues, and they are located at the mtSSB subunit region, which is essential for higher structure formation. The residue Arg38 is not clearly active in subunit assembly; however, this residue is stoichiometrically adjacent to Arg107 in the tertiary structure (being adjacent on an antiparallel β-sheet). Alteration of this residue and sidechain may, therefore, alter hydrogen bonding potential in the domain that may limit the ability of Arg107 to form stable hydrogen bonds with its neighboring isoleucine or phenylalanine residues on the opposing subunit. Two other variants, p.(Gly112Glu) and p.(Ile132Val), affect residues also located within the β-barrel scaffold, although protein modelling did not indicate hydrogen bonds formed between either of these residues and the opposing dimer. Previous functional assays showed that p.(Ile132Val) is likely to affect SSBP1 multimerization, and the mutant leads to lower thermostability of the tetramer.^7^ Altogether, variants leading to a loss of hydrogen bonds might lead to the destabilization of the dimer/tetramer or affect their assembly, representing an alternate model of a toxic allele and dominant disease. It is clear from *SSBP1*-disease cases and gnomAD population data that haploinsufficiency is unlikely to represent a pathogenic mechanism.

In addition, our report demonstrates evidence of variable disease expressivity in family 1 where the proband shows electrophysiological evidence of progressive and severe RGC dysfunction between the ages of 13 and 26 years, whereas the father of the proband has subclinical findings with mild structural changes within the retina and relatively mild RGC dysfunction. Interestingly, two other individuals carrying the p.(Arg38Glu) variant have been reported with normal visual acuity and an unspecified color vision abnormality.^8^ Taken together, these observations highlight the intrafamilial variability and possible decreased penetrance in *SSBP1*-disease. The mechanism of this is as yet unknown, but it is tempting to speculate that, given the potential dominant-negative disease model and random distribution of mutant/wildtype subunits during tetramer assembly, disease severity may at least in part be consequent upon the mutant versus *trans* allele expression level and variation at regulatory elements controlling *SSBP1* transcription. Based on the current study and previously reported detailed functional assays (tetramerization, thermostability, molecular dynamics, and mtDNA-binding capacity) the mechanism of *SSBP1*-disease could be explained by alteration of mtSSB–ssDNA interaction, and/or possibly the destabilization of the tetramer or both, with consequent likely secondary damage to the mtDNA.

In summary, we report seven additional patients harboring *SSBP1* variants with optic atrophy and retinal dystrophy. Our study demonstrates the highly variable retinal dystrophy phenotype, provides new evidence of intrafamilial variability and additional evidence of autosomal recessive inheritance in *SSBP1*-disease. The *SSBP1* gene should, therefore, be considered for NGS panels used to screen for isolated or syndromic optic atrophy and retinal dystrophy.
